# Reducing Duration of Antibiotic Use for Presumed Neonatal Early-Onset Sepsis in Greek NICUs. A “Low-Hanging Fruit” Approach

**DOI:** 10.3390/antibiotics10030275

**Published:** 2021-03-09

**Authors:** Ioannis Kopsidas, Grammatiki-Christina Tsopela, Nafsika-Maria Molocha, Eleni Bouza, Elisavet Chorafa, Evangelia Chorianopoulou, Vasileios Giapros, Despoina Gkentzi, Theodoros Gkouvas, Anastasia Kapetanaki, Korina Karachristou, Georgia Karavana, Eleni Kourkouni, Georgia Kourlaba, Maria Lithoxopoulou, Vassiliki Papaevangelou, Maria Polychronaki, Emmanuel Roilides, Tania Siahanidou, Evangelia Stratiki, George A. Syrogiannopoulos, Christos Triantafyllou, Maria N. Tsolia, Emmanouela Tsouvala, Theoklis Zaoutis, Nikos Spyridis, Preventing Hospital-Acquired Infections in Greece (PHiG) Investigators

**Affiliations:** 1 Center For Clinical Epidemiology and Outcomes Research (CLEO), 11528 Athens, Greece; c.tsopela@cleoresearch.org (G.-C.T.); nmolocha134@gmail.com (N.-M.M.); chorian_lilian@hotmail.com (E.C.); e.kourkouni@cleoresearch.org (E.K.); kurlaba@gmail.com (G.K.); c.triantafyllou@cleoresearch.org (C.T.); zaoutis@email.chop.edu (T.Z.); 2 Infectious Diseases Unit, 2nd Department of Pediatrics, National and Kapodistrian University of Athens (NKUA), 11527 Athens, Greece; mariantsolia@gmail.com (M.N.T.); nspyridis@hotmail.co.uk (N.S.); 3 B’ Neonatal Intensive Care Unit, “Aghia Sophia” Children’s Hospital, 11527 Athens, Greece; ebouzaat@otenet.gr; 4 Third Department of Pediatrics, Aristotle University of Thessaloniki, Hippokration Hospital, 54642 Thessaloniki, Greece; elsachorafa@hotmail.gr (E.C.); roilides@auth.gr (E.R.); 5 Neonatal Intensive Care Unit, University Hospital of Ioannina, 45500 Ioannina, Greece; vgiapros@uoi.gr; 6 Patras Medical School, University General Hospital of Patras, 26504 Patra, Greece; gkentzid@gmail.com; 7 Neonatal Intensive Care Unit, “Panagiotis & Aglaia Kyriakou” Children’s Hospital, 11528 Athens, Greece; theogkou@yahoo.gr; 8 Neonatal Intensive Care Unit, Elenas Venizelou Maternity Hospital, 11521 Athens, Greece; pednancy@hotmail.com; 9 A’ Neonatal Intensive Care Unit, “Aghia Sophia” Children’s Hospital, 11528 Athens, Greece; korina.karachristou@gmail.com; 10 Neonatal Intensive Care Unit, General Hospital of Nikaia and Piraeus ‘‘Aghios Panteleimon’’, 18454 Athens, Greece; gogo_kara@yahoo.gr; 11 Second Department of Neonatology, Papageorgiou Hospital, Aristotle University of Thessaloniki, 56429 Thessaloniki, Greece; mlithoxopoulou@yahoo.com; 12 Third Department of Pediatrics, National and Kapodistrian University of Athens, General University Hospital “Attikon”, 12462 Athens, Greece; vpapaev@gmail.com; 13 Neonatal Intensive Care Unit, Venizelio Hospital, 71409 Heraklion, Greece; polichronaki.mp@gmail.com; 14 Neonatal Unit of the First Department of Pediatrics, National and Kapodistrian University of Athens, 11528 Athens, Greece; siahan@med.uoa.gr; 15 Neonatal Intensive Care Unit, General District Hospital Athens “Alexandra”, 11528 Athens, Greece; zstratiki@gmail.com; 16 Department of Pediatrics, Faculty of Medicine, School of Health Sciences, University of Thessaly, 41334 Larissa, Greece; gasyrogiannopoulos@gmail.com; 17 Neonatal Intensive Care Unit, Neonatal Department, University General Hospital of Alexandroupolis, 68100 Alexandroupoli, Greece; emtsouvala@gmail.com; 18 Division of Infectious Diseases and Center for Pediatric Clinical Effectiveness, Children’s Hospital of Philadelphia, Philadelphia, PA 19104, USA

**Keywords:** early discontinuation, antibiotic stewardship, prolonged duration, empiric treatment, negative cultures, neonatal intensive care

## Abstract

Antibiotics are commonly prescribed in Neonatal Intensive Care Units (NICU), where stewardship interventions are challenging. Lowering antibiotic consumption is desperately needed in Greece, a country with high antibiotic resistance rates. We sought to assess the effectiveness of a low-cost and -resource intervention to reduce antibiotic use in Greek NICUs implementing a “low-hanging fruit” approach. A prospective quasi-experimental study was conducted in 15/17 public NICUs in Greece (9/2016–06/2019). The intervention selected was discontinuation of antibiotics within 5 days for neonates with gestational age ≥ 37 weeks, no documented signs or symptoms of sepsis, CRP ≤ 10 mg/L and negative cultures within 3 days of antibiotic initiation. Impact was evaluated by the percentage of discontinued regimens by day 5, length of therapy (LOT) and stay. Trends of antibiotic consumption were assessed with days of therapy (DOT) per 1000 patient-days. Overall, there was a 9% increase (*p* = 0.003) of antibiotic discontinuation in ≤5 days. In total, 7/13 (53.8%) units showed a ≥10% increase. Overall, 615 days on antibiotics per 1000 patients were saved. Interrupted time-series analysis established a declining trend in DOT/1000 patient-days relative to the pre-intervention trend (*p* = 0.002); a monthly decrease rate of 28.96 DOT/1000 patient-days (*p* = 0.001, 95%CI [−45.33, −12.60]). The intervention had no impact on antibiotic choice. Antibiotic use was successfully reduced in Greek NICUs using a “low-hanging fruit” approach. In resource-limited settings, similar targeted stewardship interventions can be applied.

## 1. Introduction

Antibiotic resistance is considered one of the most serious threats to global public health and is associated with increased morbidity, mortality and healthcare costs. Leading public health organizations around the world agree that among other actions, antimicrobial stewardship interventions can lead to the reduction of antibiotic use, a key driver in the evolution of resistance to different classes of antimicrobials [1].

Among European nations, Greece ranks highly in terms of antibiotic consumption and rates of antimicrobial resistance [2,3]. The latter is linked to notable disabilities, as well as deaths [4,5]. The burden is highest in infants (aged <1 year) and people >65 years, with an increasing trend compared to 2007 [5].

Antibiotics are the most commonly prescribed medications in Neonatal Intensive Care Units (NICUs) due to several predisposing factors associated with this group of patients, such as natural susceptibility to infections, prematurity, and birth-related complications, as well as postpartum events [6]. Variation in antibiotic use is also common among different NICUs [7], indicating the lack of robust evidence on appropriate indication for initiation, dosing and duration of therapy. Concurrently, there is sufficient evidence to support the link between broad spectrum antibiotic use and adverse outcomes like necrotizing enterocolitis [8,9]. Although blood culture is the gold standard for diagnosing neonatal sepsis, physicians often treat neonates with sterile cultures, despite increasing evidence that unnecessary or prolonged regimens can be harmful [10]. This represents a clear target for improvement in antibiotic use, as it has been estimated that antibiotics for culture-negative sepsis are consumed at 10 times the rate of culture-proven sepsis [11,12].

Initiating antibiotic therapy in neonates is often driven by personal judgement rather than identifying or excluding infection following practice guidelines [12], indicating that stewardship interventions could potentially focus on more straightforward objectives (“low-hanging fruit approach”) [13,14] such as stopping antibiotics early. Culture-negative early-onset sepsis is a factor contributing to high antibiotic consumption in NICUs [15].

The primary aim of this study was to assess the effectiveness of a structured intervention in order to reduce duration of antimicrobial use for culture-negative early-onset sepsis across Greek NICUs. The intervention was based on a “low-hanging fruit” approach of antibiotic stewardship practices, so that participating units could enroll patients using the existing medical personnel and no additional financial resources. A positive outcome could be an indication that low-cost initiatives can have a significant impact on prudent antibiotic use in different settings such as NICUs.

## 2. Results

A total of 1025 cases of neonates that met the inclusion criteria were identified; 507 in the pre- and 518 in the post-intervention period. Demographic characteristics and risk factors of neonates included in both study periods are listed on Table 1. The majority of them were babies delivered by caesarean section, with unknown maternal GBS status and without prolonged rupture of membranes and no chorioamnionitis.

### 2.1. Impact on Length of Therapy and on Discontinuation in 5 Days or Less

**Figure 1:**
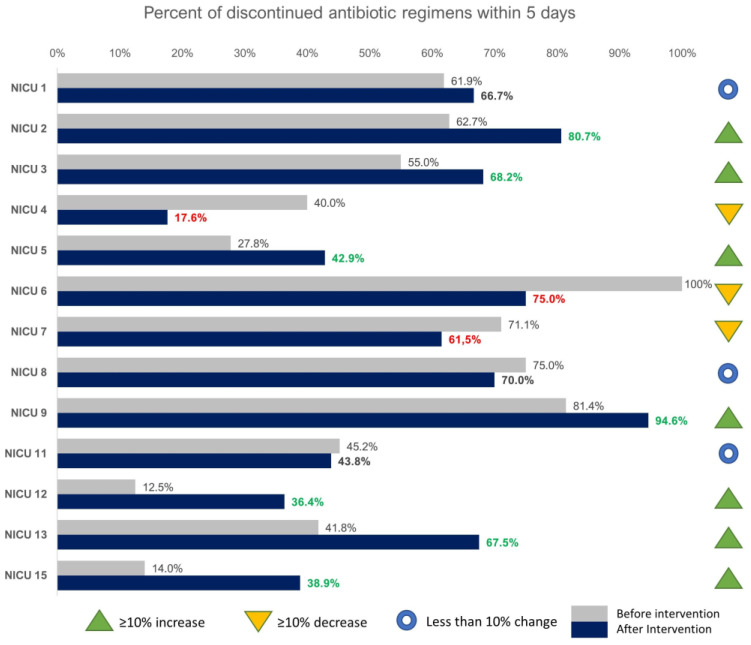
Percent of neonates in each unit that met inclusion criteria and discontinued antibiotics within 5 days of initiation of empiric treatment.

Pre-intervention data showed considerable variation in LOT for these neonates, with the median duration of antibiotic administration ranging from 2 days (IQR 2–3) to 7.5 days (IQR 6–10) across the units (Table 2). Thirteen out of the 15 units continued into the intervention phase. There was a 9% increase (*p* = 0.003) in the number of neonates that received antibiotics for five or less days in the post intervention period; from 52.5% (266/507) in the pre- to 61.5% (319/518) in the post-intervention period. Examining the changes in each unit separately (Figure 1), there was a ≥ 10% increase in discontinued regimens by day 5 in 7/13 (53.8%) of the units. Nonetheless, in three units, a ≥ 10% decrease was observed in discontinued regimens in the post-intervention period (Figure 1). Overall, 615 days of antibiotics per 1000 patients were saved during the 15 months of the post-intervention period.

**Figure 2:**
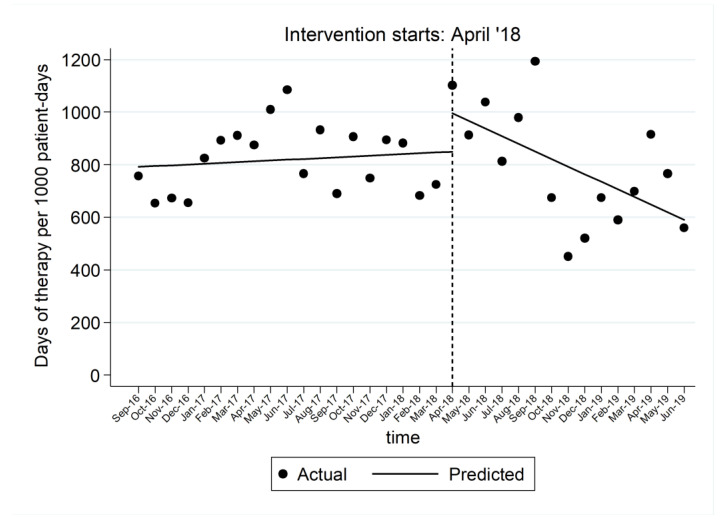
Interrupted time-series analysis of antibiotic use during the study in 13 NICUs in Greece. The period from September 2016 to March 2018 represents the pre-intervention period and April 2018 to June 2019 the post-intervention period.

The interrupted time series analysis established no significant trend prior to intervention (*p* = 0.535). However, in the post-intervention period, a decline trend was observed in the DOT/1000 patient-days relative to the pre-intervention trend (*p* = 0.002), leading to a monthly decrease rate of 28.96 days of therapy/1000 patient-days (*p* = 0.001, 95% CI = [−45.33, −12.60]) (Figure 2).

### 2.2. Prescribing Patterns, Length of Stay and Mortality

The interventions did not seem to alter the prescribing patterns in the units with regard to antibiotics selected for the treatment of EOS. For the cases that empiric treatment was discontinued within 5 days, ampicillin and aminoglycosides constituted more than 90% of the DOTs administered on the first day and throughout the course, both before and after the interventions.

Regarding the median length of stay in the seven units that discontinued antibiotics early, in two units there was a statistically significant decrease by 2 days (*p* < 0.001, *p* = 0.043). In four units the median LOS decreased by 1 to 3 days, and in one unit the median LOS increased by 1 day.

Finally, in the seven units that increased discontinuation by day 5, there were two deaths recorded among 292 cases in the pre-intervention period (6.8 deaths/1000cases) compared to one death among 309 cases (3.2 deaths/1000 cases) in the post-intervention period.

## 3. Discussion

Antibiotics are frequently prescribed in neonates for suspected EOS, even though the real risk is low. In view of antibiotics’ adverse outcomes and increased risk for infection with multidrug-resistant pathogens, antimicrobial stewardship in the NICU is important. In a country with high overall antimicrobial use and resistance rates, stewardship initiatives are needed immediately, and at the same time they need to be adapted to work in a resource-limited healthcare system. The main study findings indicate that an antibiotic stewardship intervention using a “low-hanging fruit approach” can reduce the length of antibiotic therapy in low-risk neonates treated for possible EOS in Greek NICUs. A simple intervention, in which data on antibiotic use were shared with medical staff and a goal was established to stop antibiotic therapy by day 5, led to an overall reduction of 615 antibiotic days/1000 neonates within 15 months. Interrupted time series analysis revealed a declining trend in antibiotic consumption in the post-intervention period. Participating units that increased discontinued antibiotic regimens by day 5 showed a moderate but statistically significant increase in the actual number of neonates that received ≤5 days of antibiotics in the post intervention period from 52.5% before to 61.6% after the intervention (*p* = 0.003).

Due to differences in methodology in the available literature, it is difficult to compare our results with similar studies involving stewardship interventions in NICUs [16]. Existing studies were mostly performed in single NICUs, recruited mixed populations (some including both EOS and late onset sepsis [11], and did not select comparable inclusion criteria or outcome measures.

A variety of known stewardship methodologies have been used in previous efforts to promote judicious antimicrobial use in NICUs. Revision or introduction of guidelines, prospective audit and feedback, pre-authorization, automatic stop orders and multidisciplinary rounds [11,17,18,19,20,21,22,23,24,25,26], have all been successful in lowering consumption according to the researchers’ targets. Nonetheless, in some cases, stewardship interventions do not lead to shorter duration of therapy, even if additional diagnostics are used [27]. Successful stewardship initiatives are often supported by multidisciplinary teams consisting of pharmacists, infectious diseases specialists and microbiologists [28,29,30]. In this multicenter study, resource-demanding stewardship methods were not an option, and the support by dedicated multidisciplinary teams in each center was impossible. Consequently, the targets chosen were on a higher level, and followed a low-hanging fruit approach, with the main leverage for change being the periodic reporting of antimicrobial use per unit and benchmarking with other units.

Successful reduction in the use of specific targeted agents such as vancomycin, meropenem or cefotaxime has also been documented in the literature for antimicrobial stewardship efforts in NICUs [17,18,19,31,32]. This study did not have such an aim, and as such, similar results were not identified. There was a concern that the clinicians could adopt a more aggressive prescribing pattern in view of early discontinuation. However, agents used for empiric treatment did not change during the study period, as almost all units used exclusively ampicillin with an aminoglycoside as per guidelines [33].

Duration of empiric treatment for possible EOS in the NICUs showed significant variability This is actually a common finding that has been previously reported [7,12,15,34]. The median duration of antibiotics ranged from 2 to 8 days in our population. One previous study estimated a median of 7 days and a range of 5-14 days for cases of pneumonia, despite sterile cultures and cases of culture-negative sepsis [11]. Furthermore, in a cohort of clinically well infants who were feeding by 24 h of life, duration of treatment ranged from 1 to 10 days; 11.6% of them received antibiotics for 7–10 days even though they had negative cultures and regardless of risk factors [35].

Unnecessary exposure to extended courses of antibiotic regimens in NICUs is common practice, despite good evidence that symptoms encountered in neonatal sepsis have several mimickers [6]. Stewardship interventions leading to profound declines in overall antibiotic use (up to 27%), even when including all admissions, have previously been described. In this case a 48 h electronic “hard stop” of antibiotics embedded in the electronic health record was used [11]. This is a clear indication that the magnitude of improvement in antibiotic use is linked to the organization and resources applied in ASPs. In the present study, the intervention was used in an environment of high antibiotic use, with restricted resources and a favorable outcome could be the stepping stone for further initiatives.

Why the “low-hanging fruit approach”?

For the purposes of this study, it was decided to intervene in a group of patients that were given antibiotics without appropriate indication according to national and international practice guidelines and failed to stop within 48 h. Although most of this study’s findings in terms of antibiotic overuse are relevant to countries with similar prescribing characteristics, the idea of identifying an achievable initial target before proceeding to other interventions, is applicable to all settings. The term “low-hanging fruit approach” refers to a selection of interventions that can be successful with limited resources and are easily attainable. This could involve switching antibiotics from intravenous to oral administration, stopping antibiotics early, or finding the common diagnosis linked with antibiotic overuse and developing a clinical pathway [14].

As antibiotic stewardship interventions require significant resources, complex organization and infrastructure, a full-scale program is often difficult to develop, especially in institutions where a dedicated team has not been established. In this context, choosing an easily achievable target such as establishing standardized, shorter antibiotic courses could lead to further interventions and successful outcomes. The unique environment of an intensive care unit is also important to consider. Neonatologists have a low threshold for obtaining cultures and starting antibiotics when they feel it is clinically relevant [9]. Taking into account this characteristic, this approach was chosen as a simple, feasible first goal that would also allow us to save resources and expand the program nation-wide.

Despite the limitation of automated data collection due to the lack of electronic health records, we managed to establish a national surveillance mechanism of antibiotic use in NICUs and to produce comparable data that allowed for benchmarking and identification of improvement targets.

This study has several limitations. First, we cannot be certain of the amount of blood drawn for the blood cultures taken, and we cannot account for variations in practice among units. Data on antibiotic consumption was collected in the NICUs using DOTs only for the first 7 days since the initiation of empiric treatment, while simultaneously measuring total length of therapy for each case. In the context of this study, the 7-day DOT approach could underestimate the effects of the intervention. Finally, it was not possible to measure readmission rates as a secondary outcome following our intervention, since after discharge, neonates may return to hospital on the general pediatric wards and not necessarily their local hospital.

Despite these limitations, a significant reduction of antibiotic therapy practices was documented within the network. Existing literature for this population suggests that further interventions can be applied [33,36]. However, these initial benefits could be reversed with time if sustainability of surveillance data collection and stewardship efforts cannot be ensured; a known trend previously described in pediatric antimicrobial stewardship initiatives [37]. Through this work, awareness has been raised for the need and importance of a collaborative network that collects benchmark data for quality improvement initiatives. This type of network has the potential to lead to a prolonged support of these efforts until more resources can be identified.

## 4. Materials and Methods

### 4.1. Study Design and Population

A nationwide prospective quasi-experimental study was performed, where 15 out of 17 public NICUs of the Greek National Health System contributed data between September 2016 and June 2019 after receiving ethics approval from their local authorities. Demographic, clinical, laboratory, and antibiotic consumption data were captured in an online database during the study period. Data were validated using automated algorithms and contact with the participating units when needed.

During the pre-intervention period (Sep 2016–Mar 2018), participating NICUs were asked to report the first 15 antibiotic regimens given each month, including those given within the 72 h of life for presumed early-onset sepsis (EOS), in order to explore possible targets for improvement. After reviewing these data, a group of neonates that were given prolonged antibiotic courses without sufficient risk factors for infection was identified. Neonates with the following characteristics formed the target group for the intervention: gestational age ≥37 weeks, no evidence of clinical sepsis, CRP ≤10 mg/L during the first 72 h of life and negative cultures obtained within the first 3 days of antibiotic administration.

### 4.2. Intervention

In April 2018, participating NICUs received a complete analysis of the data collected and agreed to set the goal of antibiotic discontinuation within 5 days for neonates fulfilling the characteristics mentioned above. Two units decided not to move into the intervention phase. During the post-intervention period (April 2018–June 2019), data collection was adjusted to capture all cases treated for possible EOS. There were no other exclusion criteria, nor was it expected for participating units to change their practice or conduct specific lab exams at set time points. Additionally, it was up to the physicians’ clinical judgement to decide if they would stop the antibiotics.

### 4.3. Evaluation of Impact 

To evaluate the impact of the intervention for each unit, the length of therapy (LOT) (the number of days the neonate was receiving at least one antibiotic) was calculated, as well as length of stay (LOS) for the selected cases and the percentage of discontinued antibiotic regimens by day 5. Additionally, the mean length of therapy was calculated and used to estimate the gain or loss of antibiotic days by multiplying the difference of the means before and after the intervention with the number of neonates that met the criteria in the post-intervention period. The network’s overall change in consumption was expressed per 1000 neonates for the post-intervention period. Days of therapy (DOT) per 1000 patient-days were used to assess trends of antibiotic consumption. DOT was defined as the aggregate sum of the days of exposure to each antibiotic on a 7-day follow-up from the initiation of empiric treatment. Each antibiotic for each day administered contributed by 1. For example, a neonate that was on ampicillin and gentamicin for 5 days would have an LOT of 5 days, but a DOT of 10, as each of the two antibiotics was given for 5 days. Death before discharge was also followed up through medical records. Our manuscript follows SQUIRE 2.0 guidelines [38].

### 4.4. Statistical Analysis

Categorical data are presented in absolute and relative (%) frequencies, while continuous data are presented with mean, standard deviation, median and interquartile range (IQR). Chi-square tests of independence were used to compare demographic characteristics before and after the intervention period, as well as the Mann–Whitney test, since normality of continuous data did not hold (tested with histograms). The Mann–Whitney test was also performed to compare the length of stay of neonates pre- and post-intervention (non-normal distribution). Interrupted time series analysis was used to establish whether there was a change in trend of antibiotic consumption after the implementation of the intervention. Results are presented as β-coefficients and 95% confidence intervals of the antibiotic use rate change. Statistical significance (a) was set to 5%. All analyses were performed with STATA v.13.

## 5. Conclusions

Protecting neonates from prolonged and unnecessary antimicrobial exposure constitutes a public health priority. Fully developed stewardship interventions require multiple resources in terms of personnel and financial support making them difficult to implement. Adapting stewardship practices to local needs improves outcome and encourages practicing teams to participate. Following a “low-hanging fruit approach”, a significant reduction in antibiotic use in a large network of NICUs was achieved. Implementing similar low-cost and low-resource actions could be successful in settings of high antibiotic consumption.

## Figures and Tables

**Table 1:** Demographic and clinical characteristics, maternal and neonatal, in the pre- and post-intervention period.

Number of Neonates ^&^	Pre-Intervention	Post-Intervention	*p*-Value
	507	518	
**Sex**	**N (%)**	**N (%)**	
Male	311 (61.3)	329 (63.5)	0.473
Female	196 (38.7)	189 (36.5)	
**Delivery**			
Vaginal	145 (28.7)	144 (27.9)	0.790
Caesarean	361 (71.3)	372 (72.1)	
**Group B Streptococcus status**			
Negative	151 (29.8)	132 (25.6)	0.803 *
Positive	20 (4.0)	16 (3.1)	
Unknown	335 (66.2)	367 (71.3)	
**Chorioamnionitis**			
No	476 (94.1)	413 (80.3)	0.849 *
Yes	4 (0.8)	3 (0.6)	
Unknown	26 (5.1)	98 (19.1)	
**Rupture of Membranes (>18 h)**			
No	461 (91.1)	393 (97.3)	0.194 *
Yes	21 (4.2)	11 (2.7)	
Unknown	24 (4.7)	0 (0.0)	
	**Median (IQR)**	**Median (IQR)**	
**Gestational Age (weeks)**	38 (37–39)	38 (37–39)	0.413
**Birth Weight (grams)**	3100 (2755–3420)	3140 (2800–3420)	0.275

^&^: neonates started on empiric antibiotics during the first 3 days of life, with a gestational age ≥ 37 weeks, no documented signs or symptoms or CRP ≥ 10 mg/L during the first 3 days of life, and negative cultures taken within 3 days. * *p*-value represents the differences between negative and positive or yes and no.

**Table 2:** Length of therapy before and after the intervention of neonates that met the inclusion criteria and given empiric treatment for possible early-onset sepsis.

Unit	N_1_	Mean_1_ (SD)	Median (IQR)	N_2_	Mean_2_ (SD)	Median (IQR)	Difference of Mean AB Duration before and after the Intervention	Calculated total Difference of Antibiotic Administration Days *
**NICU 1**	21	5.7 (4)	5 (3–6)	12	4.6 (1.7)	5 (3–5)	−1.1	−13.2
**NICU 2**	51	5.5 (3.4)	4 (3–7)	88	4.2 (1.5)	4 (3–5)	−1.3	−114.4
**NICU 3**	20	5.9 (2.3)	5 (4–7)	22	5.5 (2.4)	5 (4–6)	−0.4	−8.4
**NICU 4**	30	7.8 (6.9)	7 (5–9)	17	6.4 (1.8)	8 (5–9)	−1.4	−23.8
**NICU 5**	18	10.5 (8.4)	7 (5–10)	21	7.6 (3.6)	6 (5–11)	−2.9	−60.9
**NICU 6**	10	2.6 (1)	2 (2–3)	12	3.6 (1.3)	3 (3–4.5)	1	12
**NICU 7**	38	5 (2.4)	4 (3–6)	65	5.1 (2.6)	5 (3–7)	0.1	6.5
**NICU 8**	32	4.4 (1.8)	4 (3–5.5)	30	5.1 (3.6)	4 (3–7)	0.7	21
**NICU 9**	70	4.4 (3.1)	4 (3–5)	37	3.4 (1.7)	3 (2–4)	−1	−37
**NICU 10 ^**	55	5 (1.8)	5 (3–6)	−	−	−	−	−
**NICU 11**	84	7.7 (5.6)	7 (4–10)	73	8.7 (6.8)	7 (5–10)	1	73
**NICU 12**	16	7.8 (2.2)	7.5 (7–9.5)	11	8 (3.3)	6 (5–9)	0.2	2.2
**NICU 13**	67	6.4 (2.7)	6 (5–7)	77	5.1 (2.3)	4 (3–6)	−1.3	−100.1
**NICU 14 ^**	43	4.7 (2.6)	4 (3–5)	−	−	−	−	−
**NICU 15**	50	8.7 (4.2)	7.5 (6–10)	54	7.3 (3.9)	6 (5–7)	−1.4	−75.6
**Total**	605	6.2 (4.2)	5 (4–7)	518	5.8 (3.9)	5 (3–7)		−318.7

Mean_1_: Mean length of therapy before intervention. Mean_2_: Mean length of therapy after Intervention. * (Μean_1_-Μean_2_) × number of neonates meeting intervention criteria in the post-intervention period (N_2_). IQR: Inter-quartile range. ^ unit did not proceed to the intervention phase. AB: antibiotic.

## Data Availability

The datasets generated and/or analyzed during the current study are being used for further research and are available from the corresponding author on reasonable request.
